# Improving physical and mental health in women with breast cancer undergoing anthracycline-based chemotherapy through wearable device-based aerobic exercise: a randomized controlled trial

**DOI:** 10.3389/fpubh.2024.1451101

**Published:** 2024-09-19

**Authors:** Hongmei Li, Die Sang, Lijing Gong, Boliang Wang, Yong Wang, Xiao Jia, Jingjing Yu, Zhenxing Kong, Haiyun Liu, Yimin Zhang

**Affiliations:** ^1^College of Physical Education, South-Central Minzu University, Wuhan, Hubei, China; ^2^Key Laboratory of Sports and Physical Fitness Health of Ministry of Education, Beijing Sport University, Beijing, China; ^3^Department of Breast Medicine, Sanhuan Cancer Hospital, Beijing, China; ^4^Discipline of Exercise and Sports Science, Sydney School of Health Sciences, Faculty of Medicine and Health, The University of Sydney, Sydney, NSW, Australia; ^5^Department of Ultrasound, Cancer Hospital of Chinese Academy of Medical Sciences, Beijing, China

**Keywords:** wearable device, aerobic exercise, breast cancer, physical fitness, mental health

## Abstract

**Purpose:**

Aimed to assess the impact of wearable device-based aerobic exercise on the physical and mental well-being of women with breast cancer (BC) undergoing chemotherapy.

**Methods:**

Forty adult women with BC who underwent anthracycline-based chemotherapy were randomly allocated to the exercise group (*n* = 21) or the control group (*n* = 19). Both groups received standard health education and oncology care. In addition, the exercise group wore wearable devices to engage in moderate to high-intensity (50–90% HRmax) aerobic exercise during chemotherapy, while the control group did not carry out exercise intervention. Health-related physical fitness level, physical activity energy expenditure (PAEE), anxiety and depression scores, sleep quality, cancer-related fatigue, and overall quality of life (QoL), were assessed both before (prior to the first chemotherapy session) and after (prior to the fifth chemotherapy session) the exercise intervention. Exercise-related adverse events, exercise compliance, number and severity of gastrointestinal reactions and myelosuppression occurred were recorded during the exercise intervention.

**Results:**

After the intervention, compared to the control group, the exercise group (1) had significantly higher relative VO_2_peak (*p* = 0.003) and handgrip strength (*p* < 0.001); (2) had significantly higher PAEE (*p* < 0.001); (3) had a significantly lower scores in anxiety (*p* = 0.007), depression (*p* = 0.028), sleep quality in domains of subjective sleep quality (*p* = 0.010), sleep disturbances (*p* = 0.004), daytime dysfunction (*p* = 0.007), cancer-related fatigue in domains of physical (*p* < 0.001) and affective (*p* < 0.001); and (4) had a significantly lower scores in QoL in domains of physical well-being (*p* < 0.001) and emotional well-being (*p* = 0.019), while a significantly higher scores in functional well-being (*p* < 0.001). Patients in the exercise group experienced less severe gastrointestinal reactions (*p* = 0.028) and myelosuppressive symptoms (*p* < 0.001) than that in the control group. Patients in the exercise group had no serious exercise-related adverse events, with a mean exercise adherence of 81.8%.

**Conclusion:**

Wearable device-based aerobic exercise during chemotherapy can be an effective adjunctive therapy to improve physical and mental health in BC patients.

**Clinical trial registration:**

https://www.chictr.org.cn/showproj.html?proj=200247, Identifier: ChiCTR2300073667.

## 1 Introduction

In 2022, breast cancer ranked the most common cancer among women (1). In China, the estimated number of new cases and deaths from female breast cancer in 2020 were 420,000 and 117,000, respectively, ranking first in the world (2). Chemotherapy, commonly used as an adjuvant therapy for breast cancer, frequently leads to side effects such as reduced physical activity and deterioration in health-related physical fitness (3, 4). Additionally, it can result in adverse consequences such as anxiety, depression, cancer-related fatigue, sleep disturbances, gastrointestinal reactions, and myelosuppression (3, 4).

Studies have shown that breast cancer patients typically experience weight gain (1.4 ~ 6.2 kg) and negative changes in body composition during chemotherapy (5–7), often as a result of the patient’s lack of physical activity (8). Weight gain during chemotherapy for breast cancer may negatively impact quality of life (QoL), health, and self-esteem and, in severe cases, increase the risk of disease recurrence and worsen prognosis (6). Chemotherapy also leads to a significant decrease in peak oxygen uptake (VO_2_peak) (9–11), one of the indicators of cardiorespiratory fitness (CRF), with a relative VO_2_peak ≤ 18.0 mL/kg/min indicating increased risk of heart failure and functional disability (10, 12, 13). For newly diagnosed breast cancer patients, there is a correlation between health-related physical fitness and QoL. In particular, the lean body mass, VO_2_peak, and grip strength are positively associated with QoL, while body weight, BMI, body fat mass, and body fat percentage are negatively correlated (14).

Cancer-related fatigue associated with chemotherapy exacerbates both grip strength loss and weight gain (15), with grip strength being correlated with emotional issues such as anxiety and depression (16). Studies have demonstrated that cancer-related fatigue, anxiety and depression, lower levels of physical activity, and poorer physical function have been shown to be associated with sleep disturbances in breast cancer patients (17–19). Additionally, gastrointestinal reactions and myelosuppression, common side effects of chemotherapy, may lead to cancer treatment interruption. In conclusion, the above side effects from chemotherapy can seriously affect the patient’s QoL and anti-tumor confidence.

Wearable devices, such as smart bracelet, have emerged as valuable tools to enhance physical activity levels and overcome barriers to intervention, particularly in addressing cancer-related fatigue, by enabling individuals to self-supervise their activity in real time during exercise (20, 21). Supervised or partially supervised exercise training is more effective than unsupervised training in improving anxiety, QoL, and physical function (4). The study by Foulkes et al. (22) showed that wearable devices appear to improve patient exercise compliance, which is crucial for ensuring the effectiveness of exercise interventions. Previous research by our team has demonstrated that wearable devices-based moderate to high-intensity aerobic exercise could improve exercise compliance and mitigate cardiotoxicity in breast cancer patients undergoing chemotherapy (23).

Exercise is widely recognized as an effective strategy for managing breast cancer patients throughout the chemotherapy. Aerobic exercise has been shown to improve CRF (24). However, the effects of exercise on depression in cancer survivors are varied across studies (25, 26), and there is insufficient evidence regarding its influence on sleep quality, gastrointestinal response, myelosuppression, and the QoL in domains other than physical functioning in oncology patients undergoing chemotherapy (4). Therefore, this study aimed to investigate the efficacy of moderate to high-intensity aerobic exercise, facilitated by wearable devices, in improving the physical and mental well-being of women with breast cancer undergoing chemotherapy. Additionally, the safety and adherence of aerobic exercise during chemotherapy was assessed.

## 2 Materials and methods

### 2.1 Study design

This study was a randomized controlled trial in accordance with the Declaration of Helsinki, approved by the Ethics Committee of Sanhuan Cancer Hospital (SH-2022007), and written informed consent was obtained from all patients.

### 2.2 Subjects

Female patients diagnosed with stage I–III breast cancer, aged 30–65 years, who were receiving treatment at Sanhuan Cancer Hospital between March 2022 and January 2023, and scheduled for anthracycline-based chemotherapy were eligible for inclusion. Patients with absolute contraindications to exercise, as well as those with known severe structural heart disease, or pulmonary, neurologic, respiratory, or renal defects, were excluded. Participants in our studies received chemotherapy every 2 to 3 weeks with either (1) anthracycline combined with cyclophosphamide for 4 cycles and/or sequential paclitaxel for 4 cycles, or (2) anthracycline combined with paclitaxel for 6 cycles. Exclusion criteria included absolute contraindications to exercise, severe structural heart disease, cognitive impairment, or pulmonary disease. VO_2_peak is an important indicator of CRF, which is the fifth clinical vital sign (10, 27). The sample size calculation was based on an effect size for VO_2_peak of a previous study with power of 0.80 and alpha of 0.05 (11), a total of 34 patients was needed. Allowing for attrition of 17% based on our preliminary trail, ≥41 patients were considered.

### 2.3 Randomization and blinding

Following baseline measurements, participants were randomly allocated to either the exercise or control group in a 1:1 ratio using block randomization with a block size of 4. This allocation was performed by an independent researcher utilizing a computer-generated, random number sequence. Although the exercise intervention instructors were not blinded to group assignment due to their role in supervising the exercise intervention, the medical staff, outcome assessors, and participants in both groups remained unaware of the grouping throughout the study.

### 2.4 Intervention

Patients in both groups received standard health education and oncology care, encompassing health education on self-care, diet, psychological adjustment, and movement of the upper limb on the operated side. During each chemotherapy cycle, a health education lecture is given while in the hospital, and when the patient is at home, it is given via live streaming, guided by a rehabilitation therapist. Additionally, the exercise group underwent an exercise intervention, while the control group was informed of the availability of individualized exercise instruction until the completion of chemotherapy. The exercise programs were developed based on systematic reviews and clinical trials focused on exercise for the prevention and treatment of cardiotoxicity, the outcomes of cardiotoxicity that we reported in other article (23, 28).

**Figure 1:**
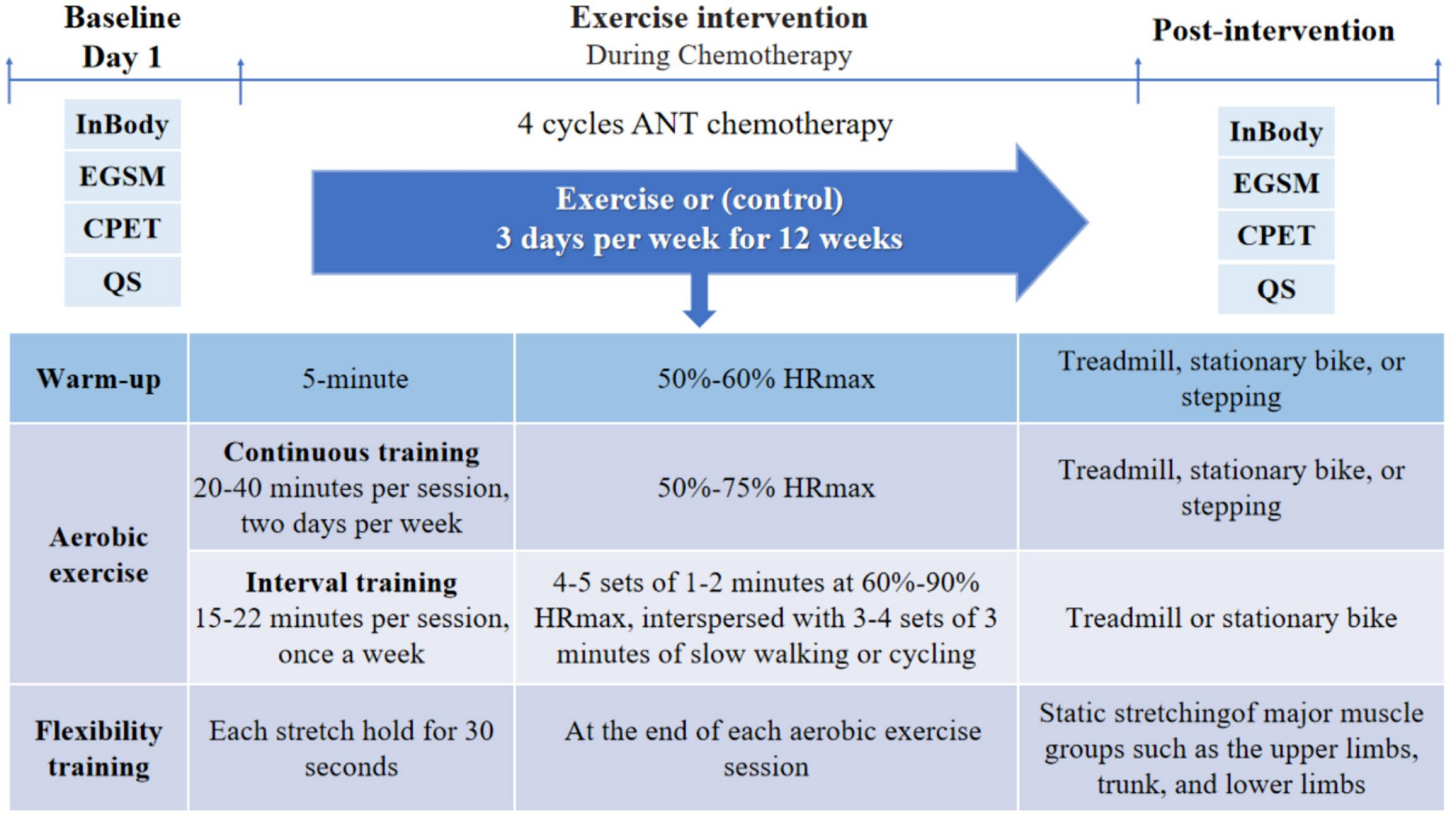
Exercise intervention protocol and times of outcome measurements. EGSM, electronic grip strength meter; CPET, cardiopulmonary exercise test; QS, questionnaire; ANT, anthracycline.

Exercise commenced concurrently with the first cycle of chemotherapy, and participants followed an individualized and progressive training regimen throughout the chemotherapy period, exercising on 3 days per week for 12 weeks during 4 cycles of anthracycline-based chemotherapy. Exercise interventions were described in previous study (23). Each exercise session consisted of a warm-up, aerobic exercise, and flexibility training (Figure 1). Prior to aerobic training, participants engaged in a 5-min warm-up at an intensity targeting 50–60% of their maximal heart rate (HRmax) through walking or cycling. Aerobic exercise encompassed both continuous and interval training. Continuous training involved treadmill, stationary bike, or stepping exercises performed at an intensity of 50–75% HRmax for 20–40 min per session, on 2 days per week. Interval training was conducted on a treadmill or stationary bike, comprising 4–5 sets of 1–2 min at 60–90% HRmax, interspersed with 3–4 sets of 3 min of slow walking or cycling, totaling 15–22 min per session, once a week. The exercise intensity increased by 5–10% HRmax and/or the exercise duration increased by 2–5 min every 3 weeks depending on the patient’s subjective physical status. Given limitations in accurately determining HRmax, the formula “HRmax = 207–0.7 × age” was utilized to establish exercise intensity for participants of all ages and fitness (29). Flexibility training was performed at the end of each aerobic exercise session, involving static stretching of major muscle groups such as the upper limbs, trunk, and lower limbs, with each stretch held for 30 s.

During hospitalization, patients in the exercise group were supervised by a rehabilitation therapist, while at home, they were instructed to self-supervise their exercise sessions, with remote supervision provided by researchers. All exercise sessions were prescribed and documented using a mobile WeChat app called “MicroMotion Manager,” developed by the Chinese team of “Exercise is Medicine,” along with a smart bracelet (Huawei 6, China). Patients were instructed to supervise exercise intensity using objective measures, such as heart rate, as well as subjective measures including the talk-test and rating of perceived exertion (RPE). Heart rate was maintained between 50 and 90% HRmax, while the talk-test ensured patients exercised at a level where they could speak with some breathlessness but could not sing. RPE was rated on a Borg scale of 6–20, with a target range between 13 and 16, representing “some exertion” to “a lot of exertion.” Mean exercise adherence was calculated as the average adherence across all participants, while exercise participation adherence and intensity adherence were calculated based on the actual attended sessions and the proportion of sessions achieving the target intensity, respectively.

### 2.5 Outcome assessments

All indicators were assessed before (prior to the first chemotherapy session) and after (prior to the fifth chemotherapy session) 4 cycles of chemotherapy (see Figure 1). Patients filled out questionnaires and scales, and underwent CRF tests and handgrip strength on the first day of hospitalization before (prior to the first chemotherapy session) and after (prior to the fifth chemotherapy session) the exercise intervention. Body composition was assessed in the morning of the second day of hospitalization, while patient was in a fasted state. Exercise adherence, the number and severity of exercise-related adverse events, gastrointestinal reactions, and myelosuppression, were recorded throughout the exercise intervention. The same tests were performed by the same physician before and after the intervention.

#### 2.5.1 Body composition

Height was measured using a height meter (Sidi RGZ-160, China) with an accuracy of 0.01 m. Waist circumference (0.5–1 cm above the navel) and hip circumference (from the pubic symphysis to the most convex part of the gluteus maximus muscle) were measured with a tape measure (Jimei T1541, China) in the morning, while fasting and wearing light clothing, with an accuracy of 0.1 centimeter. These measurements were performed twice, and the mean values were recorded. Body composition was analyzed using a body composition analyzer (InBody 770, Korea), including measurements of body weight (accurate to 0.1 kg), body mass index (BMI), muscle mass, body fat mass, body fat percentage, and visceral fat area.

#### 2.5.2 VO_2_peak and handgrip strength

The relative and absolute VO_2_peak were obtained by a physician using a standardized protocol and performed with a cycle ergometer (COSMED, Italy). The test protocol involved a 3-min rest period followed by cycling at a speed of approximately 55–65 rpm at 0 watts for 3 min. Subsequently, the workload was increased by 30 watts per minute until any three of the following four criteria were met: (1) oxygen consumption plateaued or exhibited a tendency to decline as power increased; (2) the respiratory exchange ratio reached approximately 1.05; (3) the heart rate approached ±10% HRmax; (4) the patient was unable to maintain the cycling rhythm for 10 consecutive seconds. Blood pressure was supervised every 2 min throughout the test, and the patient’s electrocardiogram (ECG) was continuously supervised using a 12-lead electrocardiogram.

Handgrip strength of habitual hand was assessed using an electronic grip strength meter (CAMRY EH101, China) with force measured to the nearest 0.1 kg. These measurements were performed twice, and the mean values were recorded.

#### 2.5.3 Physical activity energy expenditure (PAEE)

The Chinese version of the International Physical Activity Questionnaire Short Form (IPAQ-SF) was used to measure the patients’ physical activity over the past 7 days (30). IPAQ-SF consists of seven questions on the duration and frequency of walking, moderate-intensity and higher-intensity exercise, and sedentary time. The energy expenditure of walking, moderate and high intensity activity were 3.3, 4.0 and 8.0 MET, respectively (31). PAEE for 1 week, excluding the METs generated by the exercise intervention, was calculated as follows: 3.3 × time spent walking + 4 × time spent in moderate-intensity activity + 8 × time spent in higher-intensity activity.

#### 2.5.4 Anxiety and depression

The Self-rating Anxiety Scale (SAS) (32) and Self-rating Depression Scale (SDS) (33) were used to measure anxiety and depression, respectively, and both scales demonstrated good internal consistency (34). Each scale contains 20 items, rated on a 4-point Likert scale, where responses range from “1” (None or A little of the time) to “4” (Most or All of the time). The raw total scores for both the SAS and SDS range from 20 to 80, with higher scores indicating more severe anxiety and depressive symptoms (32–34).

#### 2.5.5 Cancer-related fatigue

Cancer-related fatigue was assessed using the Chinese version of the Cancer Fatigue Scale (CFS), which has demonstrated good reliability and validity in clinical cancer populations, with a Cronbach’s alpha coefficient of 0.86 for the total scale (35). The CFS comprises15 items, divided into three subscales: physical, affective, and cognitive. Each item is rated on a 5-point Likert scale, with scores ranging from 1 (“not at all”) to 5 (“a lot”) indicating the level of fatigue. The scores for each subscale are obtained by summing the scores of the items within that subscale. The physical fatigue subscale scores range from 0 to 28, while both the affective and cognitive subscales range from 0 to 16. The total fatigue score is the sum of the subscale scores, with a range from 0 to 60. Higher scores indicate more severe fatigue.

#### 2.5.6 Sleep quality

The Chinese version of the Pittsburgh Sleep Quality Index (PSQI) scale, with a test–retest correlation coefficient of 0.809, was used as a self-report assessment tool to evaluate sleep quality over a one-month period (36). The 18 self-assessment items of the PSQI compose seven components, including subjective sleep quality, sleep latency, sleep duration, sleep efficiency, sleep disturbances, use of sleeping medications, and daytime dysfunction, with each component scored on a scale of 0–3. The total scores of the PSQI range from 0 to 21, with higher scores indicating poorer sleep quality (36).

#### 2.5.7 QoL

The QoL was assessed by the Chinese version of the Functional Assessment of Cancer Therapy-Breast (FACT-B, V4.0), which has demonstrated good reliability with re-test correlation coefficients ranging from 0.82 to 0.89 for each component (37). The FACT-B comprises 36 items, organized into four subscales (physical, social/family, emotional, and functional) along with an additional breast cancer-specific subscale. Each item is rated on a 5-point Likert scale, ranging from 0 to 4, and the total score ranges from 0 to 144.

#### 2.5.8 Gastrointestinal reactions and myelosuppression

The frequency and severity of gastrointestinal reactions and myelosuppression were assessed by reviewing the patient’s electronic medical record and interviewing the attending physician. Gastrointestinal reactions included decreased appetite, nausea and vomiting, difficulty eating, diarrhea, and mouth ulcers. Myelosuppression was characterized by varying degrees of reduction in white blood cells, platelets, hemoglobin, and neutrophils. Both myelosuppression and gastrointestinal reactions were graded according to the classification standards set by the World Health Organization, utilizing a scale ranging from 0 to IV degree (38).

#### 2.5.9 Exercise-related adverse events

Exercise-related adverse events were recorded during the exercise intervention. These events were categorized as serious adverse events if they resulted in death, posed a life-threatening situation, necessitated hospitalization (or prolonged hospitalization), or caused sustained severe disability or incapacity.

### 2.6 Statistical analysis

Outcome analysis was conducted on the basis of modified intention-to-treat analyses (39). Continuous variables are expressed as mean ± standard error (X ± se), and categorical variables are expressed as *n* (%). Shapiro–Wilk’s and Levene’s tests were used to assess normality and homogeneity of variance, respectively. Baseline values were compared using independent *t* tests for continuous variables and chi-square tests for categorical variables (*χ*^2^). Missing data were filled in using the median value of multiple imputations (five imputations). Differences in outcome indicators between the two groups after the intervention were compared by repeated measures ANOVA using a general linear model when baseline values were not significantly different, and by analysis of covariance when baseline values were significantly different. Bonferroni correction was used for *post hoc* comparisons. Data analysis was performed using *SPSS* 25 (IBM, United States), with *p* < 0.05 considered statistically significant. The partial eta-squared ($\eta_{p}^{2}$) value was used to quantify effect size (40). The effect sizes were categorized as small ($\eta_{p}^{2}$= 0.01 to <0.06), medium ($\eta_{p}^{2}$= 0.06 to <0.14), or large ($\eta_{p}^{2}$≥ 0.14) (41).

## 3 Results

### 3.1 Patient’s basic information

**Figure 2:**
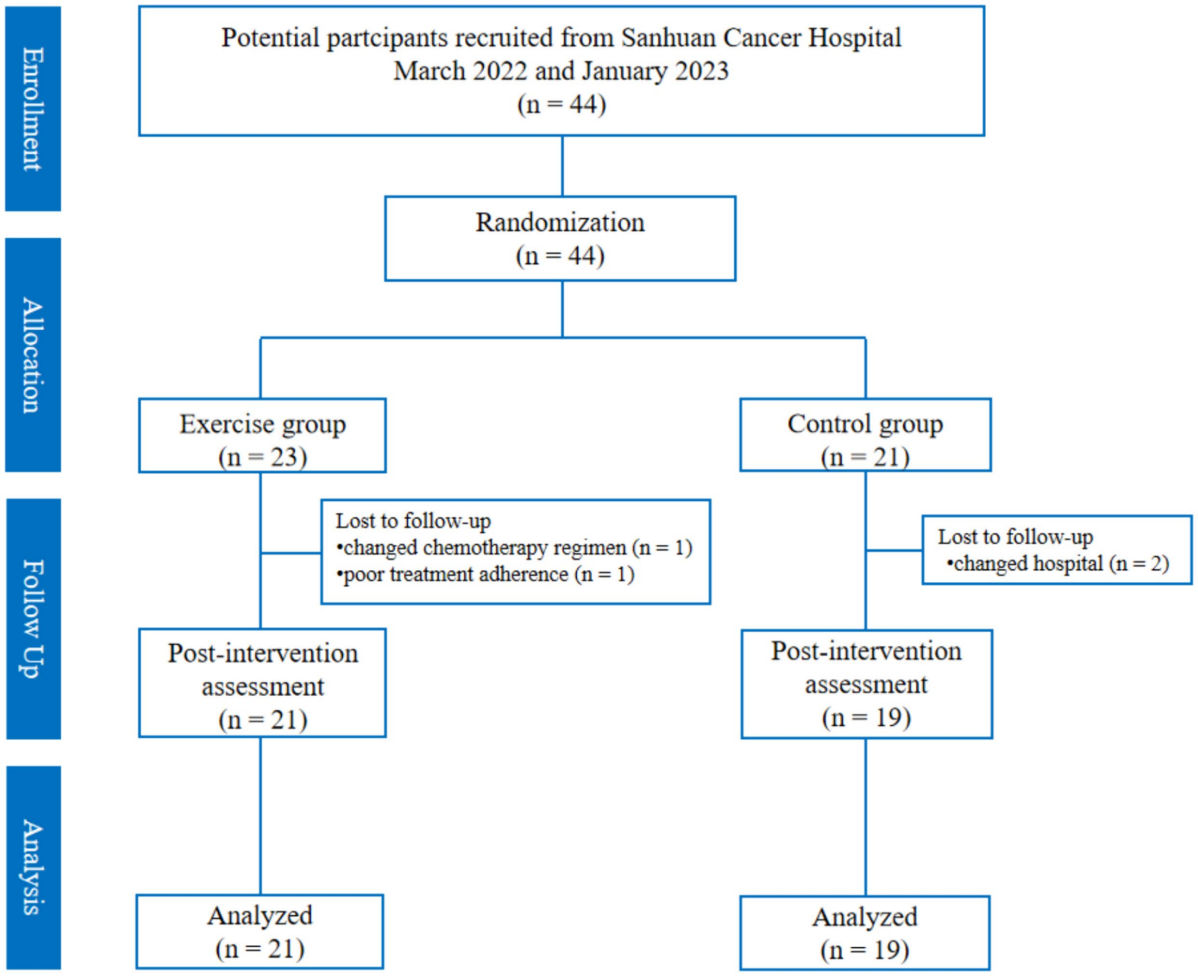
Flow of participants in the study.

The flow of participants in the study is in Figure 2. A total of 44 patients were recruited (23 patients in the exercise group and 21 patients in the control group), and a total of four patients were lost, of which two were lost because of changing hospitals for treatment (two in the control group), one changed chemotherapy regimen in the middle of the day (one in the exercise group), and one had poor adherence to chemotherapy for personal reasons (one patient in the exercise group), and finally, 40 patients were left (21 in the exercise group and 19 in the control group). There was no difference in the basic information of the lost patients in the two groups. In addition, there was no significant difference in the basic information between the control group and the exercise group (*p* > 0.05) (Table 1).

**Table 1:** Patient’s basic information.

	Control group (*n* = 19)	Exercise group (*n* = 21)	*P*
Demographics			
Age (year)	48.47 ± 2.13	47.38 ± 1.96	0.708
Height (m)	1.61 ± 0.01	1.59 ± 0.01	0.105
Weight (kg)	61.48 ± 2.13	61.93 ± 1.74	0.867
BMI (kg/m^2^)	23.48 ± 0.75	24.36 ± 0.65	0.384
Menstruation			
Premenopausal	15 (79.0%)	16 (76.2%)	0.835
Postmenopausal	4 (21.0%)	5 (23.8%)	
Concomitant			
Hypertension	3 (15.8%)	2 (9.5%)	0.550
Type 2 Diabetes	1 (5.3%)	1 (4.8%)	0.942
Hyperlipidemia	1 (5.3%)	0 (0%)	0.287
Fatty liver	4 (21.1%)	3 (14.3%)	0.574
Molecular type			
Luminal A	1 (5.3%)	0 (0%)	0.287
Luminal B	14 (73.7%)	13 (61.9%)	0.427
Luminal B HER 2 (3+)	1 (5.3%)	4 (19.0%)	0.188
Triple-negative	3 (15.8%)	4 (19.0%)	0.787
Tumor stage			
I	1 (5.3%)	1 (4.8%)	0.942
II	8 (42.1%)	8 (38.1%)	0.796
III	10 (52.6%)	12 (57.1%)	0.775
Chemotherapy			
Neo-adjuvant chemotherapy	2 (10.5%)	3 (14.3%)	0.720
Adjuvant chemotherapy	17 (89.5%)	18 (85.7%)	
Chemotherapy protocol			
AC	1 (5.3%)	1 (4.8%)	0.942
AC-T	15 (78.9%)	16 (76.2%)	0.835
AT	3 (15.8%)	4 (19.0%)	0.787
Anthracycline dosage (mg/m^2^)	494.11 ± 31.26	452.38 ± 27.89	0.324

BMI, body mass index; AC, anthracyclines combined with cyclophosphamide; AC-T, anthracycline combined with cyclophosphamide, and sequential paclitaxel; AT, anthracyclines combined with paclitaxel.

### 3.2 Changes in patients’ physical and mental health

#### 3.2.1 The health-related physical fitness

As shown in Table 2, before the intervention, there were no significant differences in the indicators between the two groups. After the intervention, a significantly higher relative VO_2_peak (+2.425 mL/kg/min, *p* = 0.003) and handgrip strength (+7.100 kg, *p* < 0.001) in the exercise group compared with the control group. The body fat mass (+1.016 kg, *p* = 0.013), body fat percentage (+1.426%, *p* = 0.009), and visceral fat area (+7.174 cm^2^, *p* = 0.011) were significantly higher, while handgrip strength (−1.247 kg, *p* = 0.017) was significantly lower after the intervention than before the intervention in the control group. The waist circumference (−3.024 cm, *p* = 0.011) and waist-to-hip ratio (−0.031, *p* = 0.003) were significantly lower, while handgrip strength (+3.062 kg, *p* < 0.001) was significantly higher after the intervention than before the intervention in the exercise group.

**Table 2:** Changes in the health-related physical fitness.

	Control group (*n* = 19)		Exercise group (*n* = 21)	
	Before intervention	After intervention	Before intervention	After intervention
Weight (kg)	61.48 ± 2.13	61.86 ± 2.20	61.93 ± 1.74	61.54 ± 1.59
BMI (kg/m^2^)	23.48 ± 0.75	23.66 ± 0.77	24.36 ± 0.65	24.20 ± 0.59
Skeletal muscle mass (kg)	22.79 ± 0.61	22.38 ± 0.59	22.45 ± 0.60	22.35 ± 0.54
Body fat mass (kg)	19.38 ± 1.57	20.39 ± 1.57^#^	20.39 ± 0.99	20.20 ± 0.95
Body fat percentage (%)	30.88 ± 1.59	32.31 ± 1.46^##^	32.56 ± 0.95	32.53 ± 0.93
Visceral fat area (cm^2^)	93.97 ± 9.41	101.15 ± 9.49^#^	98.38 ± 5.70	97.82 ± 5.58
Waist circumference (cm)	82.45 ± 2.21	83.58 ± 2.27	84.21 ± 1.83	81.19 ± 1.74^#^
Hip circumference (cm)	97.25 ± 1.26	97.66 ± 1.56	97.55 ± 1.00	96.81 ± 1.02
Waist-to-hip ratio	0.85 ± 0.02	0.85 ± 0.01	0.86 ± 0.02	0.83 ± 0.01^##^
Absolute VO_2_peak (L/min)	1.07 ± 0.05	1.03 ± 0.04	1.12 ± 0.05	1.15 ± 0.04
Relative VO_2_peak (mL/kg/min)	17.31 ± 0.77	16.29 ± 0.55	18.04 ± 0.77	18.65 ± 0.48^**^
Handgrip strength (kg)	19.22 ± 0.97	17.97 ± 0.89 ^#^	21.50 ± 1.04	24.57 ± 1.12^**, ##^

^**^p < 0.01, between-groups comparison at the same time; ^#^*p* < 0.05 and ^##^*p* < 0.01, intragroup comparison.

A *post hoc* sensitivity analysis for missing data with multiple imputations was performed by comparing the results of general linear model repeated measure ANOVA before and after multiple imputation (Table 3).

**Table 3:** Results of GLMM ANOVA before and after multiple imputation.

	F	*P*	$\eta_{p}^{2}$
Absolute VO_2_peak (before imputation)			
Group × time	2.030	0.175	0.119
Group	1.621	0.222	0.098
Time	0.673	0.425	0.043
Absolute VO_2_peak (after imputation)			
Group × time	1.607	0.221	0.082
Group	2.837	0.109	0.136
Time	0.002	0.969	<0.001
Relative VO_2_peak (before imputation)			
Group × time	2.029	0.175	0.119
Group	4.620	**0.048**	0.235
Time	0.523	0.481	0.034
Relative VO_2_peak (after imputation)			
Group × time	2.416	0.137	0.118
Group	4.705	**0.044**	0.207
Time	0.037	0.850	0.002

GLMM, general linear model repeated measure. Significant differences are marked in bold.

#### 3.2.2 PAEE

As shown in Table 4, there was no significant difference in PAEE between the two groups before the intervention. After the intervention, a significantly higher PAEE in the exercise group compared with the control group (+736.553 MET/min/w, *p* = 0.001). The PAEE was significantly lower in the control group (−122.026 MET/min/w, *p =* 0.039), while significantly higher in the exercise group (+517.738 MET/min/w, *p* = 0.001) after the intervention than before the intervention.

**Table 4:** Changes in PAEE.

	Control group (*n* = 19)		Exercise group (*n* = 21)	
	Before intervention	After intervention	Before intervention	After intervention
PAEE (MET/min/w)	325.47 ± 61.65	203.44 ± 38.55^#^	381.98 ± 73.60	899.71 ± 164.89^**, ##^

^**^p < 0.01, between-groups comparison at the same time; ^#^*p* < 0.05 and ^##^*p* < 0.01, intragroup comparison.

#### 3.2.3 Anxiety and depression

As shown in Table 5, there were no significant differences in anxiety and depression between the two groups before the intervention. After the intervention, a significantly lower scores both in anxiety (−5.737, *p* = 0.007) and depression (−7.000, *p* = 0.028) in the exercise group compared with the control group. Both anxiety (+4.368, *p* = 0.004) and depression (+4.474, *p* = 0.025) scores were higher in the control group after the intervention than before the intervention.

**Table 5:** Changes in anxiety and depression.

	Control group (*n* = 19)		Exercise group (*n* = 21)	
	Before intervention	After intervention	Before intervention	After intervention
Anxiety	32.63 ± 1.13	37.00 ± 1.49^##^	30.95 ± 1.04	31.76 ± 1.27^**^
Depression	36.26 ± 1.17	40.74 ± 2.29^#^	35.05 ± 1.22	34.57 ± 1.67^*^

^*^P < 0.05 and ^**^P < 0.01, between-groups comparison at the same time; ^#^*p* < 0.05 and ^##^*p* < 0.01, intragroup comparison.

#### 3.2.4 Cancer-related fatigue

As shown in Table 6, there were no significant differences in the score of the three subscales between the two groups before the intervention. After the intervention, a significantly lower scores both in physical fatigue (−6.105, *p* < 0.001) and affective fatigue (−3.579, *p* < 0.001) in the exercise group compared with the control group. The scores of physical fatigue (+4.158, *p* = 0.002), affective fatigue (+1.474, *p* = 0.007), and cognitive fatigue (+2.632, *p* = 0.018) were higher in the control group after the intervention than before the intervention.

**Table 6:** Changes in cancer-related fatigue.

	Control group (*n* = 19)		Exercise group (*n* = 21)	
	Before intervention	After intervention	Before intervention	After intervention
Physical fatigue	8.63 ± 1.33	12.79 ± 0.94^##^	7.24 ± 0.73	7.00 ± 0.82^**^
Affective fatigue	6.89 ± 0.46	8.37 ± 0.56^##^	5.90 ± 0.58	4.95 ± 0.55^**^
Cognitive fatigue	5.74 ± 0.71	8.37 ± 0.86 ^#^	4.95 ± 0.62	5.81 ± 0.84

^**^p < 0.01, between-groups comparison at the same time; ^#^*p* < 0.05 and ^##^*p* < 0.01, intragroup comparison.

#### 3.2.5 Sleep quality

As shown in Table 7, there were significant between group differences in sleep latency, sleep efficiency and total score before intervention. Analysis of covariance showed that there was no significant difference between the two groups after the intervention (*p* > 0.05). After the intervention, a significantly lower scores in subjective sleep quality (−0.632, *p* = 0.010), sleep disturbances (−0.632, *p* = 0.004), and daytime dysfunction (−0.842, *p* = 0.007) in the exercise group compared with the control group.

**Table 7:** Changes in sleep quality.

	Control group (*n* = 19)		Exercise group (*n* = 21)	
	Before intervention	After intervention	Before intervention	After intervention
Subjective sleep quality	1.53 ± 0.12	1.68 ± 0.13	1.19 ± 0.16	1.14 ± 0.17^*^
Sleep latency	2.00 ± 0.20	1.74 ± 0.20	1.14 ± 0.21^*^	1.24 ± 0.27
Sleep duration	1.05 ± 0.27	0.95 ± 0.21	0.71 ± 0.17	0.81 ± 0.27
Sleep efficiency	1.42 ± 0.28	1.16 ± 0.21	0.57 ± 0.16^*^	0.67 ± 0.24
Sleep disturbances	1.05 ± 0.12	1.63 ± 0.14^##^	1.00 ± 0.10	1.05 ± 0.11^**^
Use of sleeping medications	0.16 ± 0.16	0.32 ± 0.17	0.05 ± 0.05	0.29 ± 0.16
Daytime dysfunction	2.00 ± 0.34	2.05 ± 0.16	1.29 ± 0.21	1.29 ± 0.20^**^
Total score	9.00 ± 0.84	9.47 ± 0.75	5.95 ± 0.73^*^	6.57 ± 1.10

^*^p < 0.05 and ^**^p < 0.01, between-groups comparison at the same time; ^##^*p* < 0.01, intragroup comparison.

The score of sleep disturbances was significantly higher in the control group after the intervention than before the intervention (+0.597, *p* = 0.004).

#### 3.2.6 QoL

As shown in Table 8, there were no significant differences in the score of the five subscales between the two groups before the intervention. After the intervention, a significantly lower scores in physical well-being (−6.747, *p* < 0.001) and emotional well-being (−2.263, *p* = 0.019), while a significantly higher scores in functional well-being (+8.474, *p* < 0.001) in the exercise group compared with the control group. Physical health score in the control group were significantly higher after the intervention than before the intervention (+5.158, *p* < 0.001). Functional health score in the exercise group were significantly higher after the intervention than before the intervention (+2.667, *p* = 0.017).

**Table 8:** Changes in QoL.

	Control group (*n* = 19)		Exercise group (*n* = 21)	
	Before intervention	After intervention	Before intervention	After intervention
Physical well-being	5.37 ± 0.97	10.53 ± 0.85^##^	4.95 ± 0.91	4.43 ± 0.58^**^
Social/Family well-being	17.53 ± 0.75	16.63 ± 0.85	17.24 ± 1.24	19.43 ± 1.16
Emotional well-being	7.32 ± 0.84	7.53 ± 0.59	6.90 ± 0.78	5.38 ± 0.48^*^
Functional well-being	13.37 ± 1.37	10.42 ± 1.04	15.95 ± 1.06	18.62 ± 1.24^**, #^
Additional concerns	8.63 ± 1.39	11.11 ± 0.62	11.10 ± 1.32	9.33 ± 0.67
Total score	52.26 ± 3.01	55.68 ± 1.58	55.71 ± 3.15	56.71 ± 2.13

^*^p < 0.05 and ^**^p < 0.01, between-groups comparison at the same time; ^#^*p* < 0.05 and ^##^*p* < 0.01, intragroup comparison.

#### 3.2.7 Gastrointestinal reactions and myelosuppression

During the exercise intervention, patients in the control group received a total of 76 anthracycline-based chemotherapy treatments, while patients in the exercise group received a total of 84 anthracycline-based chemotherapy treatments. The occurrence of gastrointestinal reactions and myelosuppression in the patients is shown in Table 9.

**Table 9:** Occurrence of gastrointestinal reactions and myelosuppression in patients.

	Gastrointestinal reactions [cases (%)]			Myelosuppression [cases (%)]		
	I	II	III	I	II	III
Control group (*n* = 19)	5 (6.6%)	53 (69.7%)	18 (23.7%)	20 (26.3%)	45 (59.2%)	2 (2.6%)
Exercise group (*n* = 21)	7 (8.3%)	34 (40.5%)	3 (3.6%)	26 (31.0%)	9 (10.7%)	0 (0%)
*χ* ^2^	7.174			18.571		
*P*	0.028			<0.001		

#### 3.2.8 Exercise-related adverse events and exercise adherence

No serious exercise-related adverse events were observed during the exercise intervention. However, patients in the exercise group did experience non-serious events during exercise, including toe pain, knee pain, and weakness, as detailed in Table 10.

**Table 10:** Adverse events during exercise in patients in the exercise group [cases (times)].

Categorizations	Cases (times)
Toe pain	2 (8)
Knee pain	1 (1)
Weakness	12 (59)

Out of the 720 planned sessions (18 carried out 36 sessions and 3 carried out 24 sessions) for the exercise group, patients participated in 589 sessions, resulting in an average adherence rate of 81.8% (range 61.1% ~ 100%), Additionally, the average adherence to exercise cycle duration was 91.9% (range 70.0% ~ 100%), and the average adherence to exercise intensity was 92.5% (range 78.6% ~ 100%).

## 4 Discussion

The main finding of this study was that the body fat mass, body fat percentage, and visceral fat area were significantly higher after the intervention than before the intervention in the control group, while the waist circumference and waist-to-hip ratio were significantly lower after the intervention than before the intervention in the exercise group. Additionally, relative VO_2_peak and handgrip strength were significantly increased, and overall sleep quality and QOL were significantly improved, while anxiety, depression and cancer-related fatigue level significantly reduced in the exercise group compared to the control group. Notably the exercise intervention was well-tolerated, with no reports of serious adverse events among participants. Furthermore, individuals in the exercise group experienced fewer gastrointestinal reactions and myelosuppression symptoms, with overall less severe symptoms compared to the control group. These findings underscore the safety and potential benefits of wearable device-based aerobic exercise during chemotherapy for breast cancer patients. Moreover, the observed improvements in fitness levels suggest broader implications for enhancing overall health and well-being in this population.

Studies have demonstrated that patients undergoing chemotherapy for breast cancer often experience weight gain and adverse alterations in body composition, characterized primarily by an increase in fat mass without concurrent loss of muscle mass (7). This heightened adiposity elevates the risk of tumor recurrence and diminishes treatment response (7, 8). Notably, the accumulation of fat in the abdomen, particularly excessive visceral fat, is strongly linked to the onset of metabolic and cardiovascular diseases (42), thereby influencing the trajectory and efficacy of cancer treatment and heightening the risk of cancer-related mortality (43). Waist circumference and waist-to-hip ratio serve as standard indicators for diagnosing abdominal obesity and exhibit positive correlations with total visceral fat quantified through abdominal CT scans (42, 44). A key finding of this study was the significant reduction in waist circumference and waist-to-hip ratio observed in the exercise group, while the control group had a significant increase in body fat mass, body fat percentage, and visceral fat area following the intervention. This suggests that aerobic exercise may effectively prevent fat gain and improve fat distribution among breast cancer patients, thereby holding meaningful clinical significance in reducing the risk of cardiovascular disease and cancer recurrence. There were no differences in weight loss, BMI, and several indicators of body fat accumulation and distribution between the two groups, it may take more time to observe significant changes, and exercise could prevent or at least slow down the rate of growth in the negative direction.

Another important finding from our study was that relative VO_2_peak and handgrip strength were significantly increased. It is worth noting that for every additional 1 mL/kg/min increase in oxygen uptake in women, the risk of all-cause mortality decreases by 11.3% (45). Although the relative VO_2_peak in the exercise group of our study increased by 0.71 mL/kg/min, which was lower than the expected increase reported in previous research (46), the increase of 2.425 mL/kg/min in the exercise group compared with the decrease in VO_2_peak in the control group suggests that aerobic exercise was effective in preventing the decrease in VO_2_peak. Furthermore, the percentage of functional disability was lower in the exercise group compared to the control group after the intervention (35% vs. 75%). Enhanced CRF enables patients to experience reduced fatigue and dyspnea during daily and recreational activities (46). Additionally, the increased grip strength observed in the exercise group might contribute to an improved functional domain of QoL and aid in their return to routine activities (47). Patients with cancer who have higher grip strength may have better performance in daily activities and recreational tasks requiring upper extremity strength, such as towel wringing, vacuuming, gardening, shopping, and exercising (14). Moreover, greater grip strength may correlate with fewer co-morbidities and reduced surgical complications (e.g., lymphedema, muscle adhesions, and decreased mobility) (14). Finally, higher grip strength has been associated with a reduced risk of mortality. The cut-off of low handgrip strength less than 16.1 kg was associated with cancer mortality in women with breast cancer, with a hazard ratio of 1.593 (15, 48). The relative increase of 4.32 kg in the exercise group compared to the control group’s decrease in handgrip strength effectively prevented a decline in handgrip strength.

Consistent with the results of the present study, Chen et al. (49) found that a home-based exercise regimen of 3 days per week, with each session lasting 40 min, significantly reduced anxiety and depression compared to usual care treatments. The mechanisms through which exercise improves anxiety and depression encompass both biological and psychosocial factors, including the production of “pleasure” chemicals in the brain (e.g., endorphins, dopamine), neural signaling repair, reduction of inflammation, exposure to pleasant outdoor environments, social interaction, distraction from stress, enhanced self-efficacy, and increased self-esteem (50). Additionally, a review by Singh et al. (51) revealed that exercise interventions lasting ≤12 weeks were more effective in alleviating anxiety and depression compared to that lasting ≥12 weeks. This difference may be attributed to exercise adherence, so how to help patients maintain training is a challenge, highlighting the importance of selecting appropriate approaches to optimize adherence to the long-term exercise regimen.

A novel finding of this study was the observation that the exercise group experienced fewer gastrointestinal reactions and myelosuppression and exhibited less severe symptoms compared to the control group, implying that aerobic exercise might relieve the gastrointestinal reactions and myelosuppression commonly associated with chemotherapy. Myelosuppression, characterized by a decrease in leukocyte count, is a frequent side effect of chemotherapy, weakening the body’s immune system and predisposing individuals to serious infections and bleeding. Good sleep quality is crucial for immune function, as it enhances sympathetic excitability to adrenergic receptor-mediated actions in bone marrow cells and regulates the migration efficiency of bone marrow stem cells, thereby stimulating leukocyte proliferation (52). Following the intervention, the exercise group demonstrated improved sleep duration and experienced fewer sleep disturbances and less daytime dysfunction compared to the control group. Sleep quality is influenced by various factors, including physical activity level, physical functioning, fatigue, and spirituality, and improvements in these aspects may contribute to enhanced sleep quality and improvements in the physical, emotional, and functional domains of QoL.

Chemotherapeutic drug-induced vagal reflexes, leading to the gag reflex, have been associated with pentraxin receptor-3 (5-HT3) inhibition (53). However, studies have indicated that exercise functions as a 5-HT3 agonist, thus potentially alleviating depression (54). Therefore, aerobic exercise may alleviate gastrointestinal reactions through alternative pathways. Cao et al. (52) proposed that an optimistic perception gives patients a sense of control over their disease and enables effective coping with adverse effects of chemotherapy, including gastrointestinal reactions and myelosuppression. Aerobic exercise could potentially reduce patients’ perceptual stress, thereby fostering a greater sense of control over adverse effects. This may also be one of the reasons why aerobic exercise improved physical well-being and additional concerns of QoL.

No serious exercise-related adverse events occurred in patients in the exercise group of this study, indicating that is safe to perform wearable device aerobic exercise during chemotherapy. The mean exercise adherence in this study was 81.3%, which is higher than previous studies reporting of 63.2% ~ 76% for a 12-week exercise intervention (55, 56). In this study, smart wearable devices were used in this study for supervision and management to improve patients’ exercise adherence (20–22). The notable improvement in exercise adherence contributed significantly to the substantial increase in PAEE and the enhancement of physical and mental health among patients in the exercise group. However, it is unclear if it would generate additional benefits compared to aerobic exercise without wearable devices.

## 5 Limitations and suggestion

The study has several limitations, first, comparisons of the effects of exercise without and with wearable devices were not made due to the challenge of recruiting large number of patients in the clinical setting. Second, although exercise workouts were scheduled for patients three times a week, the increase in PAEE in the exercise group may be attributed to patients’ increased spontaneous walking after experiencing symptom improvement. Despite this, we continued to monitor and control the exercise regimen as part of our randomized controlled trial. Future research endeavors could focus on recruiting larger cohorts, if feasible, to explore the long-term effects of exercise in this population with precise equipment to monitor follow-up in real time.

## 6 Conclusion

In conclusion, this study demonstrates that engaging in moderate to high-intensity wearable device-based aerobic exercise for three times a week over a period of 12 weeks yields numerous benefits for breast cancer patients undergoing chemotherapy. The observed improvements in physical and mental health may empower patients, fostering a more energetic and positive mindset to combat cancer. Importantly, exercise serves as a non-side-effective, non-resistant, and cost-effective intervention compared to pharmacological treatments.

## Data Availability

The data underlying this article will be shared on reasonable request to the corresponding author.
